# Smartphone Sensors for Stone Lithography Authentication

**DOI:** 10.3390/s140508217

**Published:** 2014-05-07

**Authors:** Giuseppe Schirripa Spagnolo, Lorenzo Cozzella, Donato Papalillo

**Affiliations:** Dipartimento di Matematica e Fisica, Università degli Studi “Roma Tre”, Via della Vasca Navale 84, I-00146 Roma, Italy; E-Mails: lorenzo.cozzella@uniroma3.it (L.C.); donato.papalillo@uniroma3.it (D.P.)

**Keywords:** biometry, artworks authentication, speckle metrology, digital image processing, lithography, smartphone, mobile computing

## Abstract

Nowadays mobile phones include quality photo and video cameras, access to wireless networks and the internet, GPS assistance and other innovative systems. These facilities open them to innovative uses, other than the classical telephonic communication one. Smartphones are a more sophisticated version of classic mobile phones, which have advanced computing power, memory and connectivity. Because fake lithographs are flooding the art market, in this work, we propose a smartphone as simple, robust and efficient sensor for lithograph authentication. When we buy an artwork object, the seller issues a certificate of authenticity, which contains specific details about the artwork itself. Unscrupulous sellers can duplicate the classic certificates of authenticity, and then use them to “authenticate” non-genuine works of art. In this way, the buyer will have a copy of an original certificate to attest that the “not original artwork” is an original one. A solution for this problem would be to insert a system that links together the certificate and the related specific artwork. To do this it is necessary, for a single artwork, to find unique, unrepeatable, and unchangeable characteristics. In this article we propose an innovative method for the authentication of stone lithographs. We use the color spots distribution captured by means of a smartphone camera as a non-cloneable texture of the specific artworks and an information management system for verifying it in mobility stone lithography.

## 1. Introduction

Mobile phones and smartphones [1] have been increasing their computational power in the last years. These new devices bring together imaging, processing, communication and displaying capabilities. At the same time, these new smartphone potentialities bring new application possibilities [2].

Starting with the possibilities offered by the processing capabilities and performance of the new photo cameras, present in the new smartphones, in this paper we have developed a simple, robust, efficient and low-cost system for lithography authentication.

The main problem when we buy an artwork object consists in getting a certificate of authenticity, in particular for artwork bought through a seller and not first hand from the artist. There is a tremendous abuse in the “certificate of authenticity” business because, unless a certificate of authenticity is originated and directly signed by the artist, any other possibility such as by the publisher of the art (in the case of limited editions), a confirmed dealer or agent of the artist (not a third party or reseller), or an acknowledged expert on the artist is pretty much meaningless. A legitimate one must contain specific details about the artwork, such as when and how it was produced, the names of people or companies involved in its production, dimensions, and the names of reference books or similar resources that contain either specific or related information about either that work of art and/or the artist. It should also state the qualifications and full contact information of the individual or entity that authored the certificate, and include his or her complete and current contact information.

Unfortunately, these certificates are often cloned: the same document is supplied by the seller to certificate the originality of more than one single artwork.

Certificates of authenticity are often problematic; many are just worthless. In general, most people believe that art with a certificate is automatically genuine, but that is not even close to truth. Currently there are no laws that regulate who is authorized (or is not) to produce certificates of authenticity, or what types of statement, information or documentation a certificate of authenticity must contain. In other words, anyone can write a certificate whether or not they are qualified. As if that were not bad enough, unscrupulous sellers forge certificates of authenticity and use them either to sell outright fakes or to misrepresent existing works of art as being more important or valuable than they actually are. A possible fraud can be put the following way into effect: an art merchant, starting from an original lithograph and its original certificate of authenticity, duplicates both and sells false artwork as genuine, using false certificate of authenticity as proof of originality.

A solution to this problem would be the use of the technology offered by modern smartphones to connect to a proper website which would thus allow checking the origin of the artwork. The website is designed to contain information about the artwork and a digital certificate of authenticity. This digital certificate links information on non-cloneable features of the specific artworks. In this way the inappropriate usage will not be possible and the buyer will be able to verify the originality by himself. To do this it is necessary, for a single artwork, to find unique, unrepeatable, and unchangeable characteristics. If these characteristics are present, we have the possibility to identify the artwork and to distinguish it from another one [3–6]. By choosing the opportune characteristic, such kind of identification can be applied to many types of artwork objects.

Although lithography after World War II was generally considered a commercial medium, actually, it is an important artistic medium [7], used during the 20th century, also by a group of artists, including Braque, Calder, Chagall, Dufy, Léger, Matisse, Miró, and Picasso, who rediscovered the largely undeveloped art form of lithography. It has also to be noted, in relation to stone lithography, that only a finite and tiny number of copies is possible, because stones are ruined by the impression process, and after a certain number of reproductions cannot be used anymore. In fact, at any impression, the stone itself is degraded and after some copies it has to be destroyed and substituted. For this reason, lithography is also the most counterfeited artwork; in fact an expert can also easily make a mistake in asserting the authenticity of a masterpiece.

Lithographs have a random structure that is not cloneable (the position of color spots). The presence of these non-duplicable features enables us to develop a system capable of distinguishing two different lithographs, even when coming from the same series. Therefore, in this paper we will implement a new authentication method based on image acquisition by smartphone cameras, a web information system and a verification procedure similar to biometric identification.

In particular, the article is divided in two parts: the first one, after having described the lithographic technique to better understand why they are so easily counterfeited, presents an information management system, which allows using web distributed information for verifying lithography authenticity, using data acquired from a new Certificate of Originality and from the lithography itself. The second part describes in detail the automatic verification procedure, to be carried on the smartphone itself, based on a biometric-like approach.

## 2. Unique Characteristics Determination and Acquisition Using a Smartphone System

### 2.1. Stone Lithography

**Figure 1.**
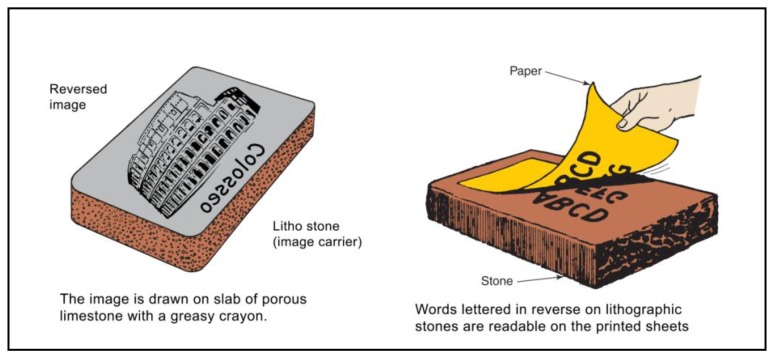
The lithography process uses a stone made of a porous material containing a reversed image. Every lithographic creation requires a pressure of the stone on the paper support, with a subsequent impact on the crayon image reported on it.

The term lithograph or lithography comes from Greek, meaning “writing with stone”. The German Alois Senefelder [8] invented it in 1798. The technical process of lithography is based on the principle that limestone is naturally attracted to oil, and that oil and water have a natural antipathy, refusing to mix each other. A simplified version of the process is the following: (1) the artist draws an image on lithographic stone with a greasy crayon; (2) the stone is moistened with water. Parts of the stone not protected by the grease soak up the watert (3) oil-based ink is rolled onto the stone. The greasy parts of the stone pick up the ink, while the wet parts do not; (4) A piece of paper is pressed onto the stone and the ink transfers itself from the stone to the paper [9,10]. Figure 1 explains the procedure described above.

**Figure 2.**
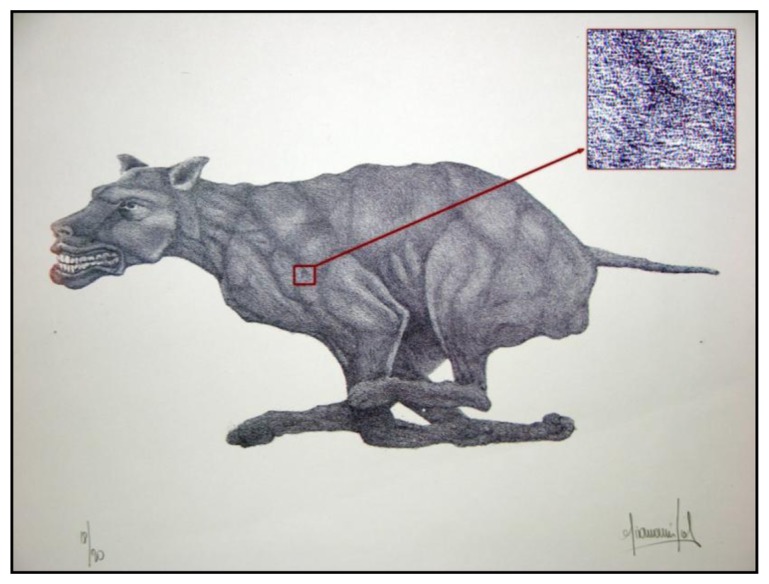
Stone lithography executed by the artist Giovanni Job (Dog 18/20).

Color hand-made lithographs require the production of a new plate (stone) for each color. It is not uncommon to print more colors, so the artist can become involved in a long process of production. Figure 2 shows an example of stone lithography made by the artist Giovanni Job using the above-described method.

Every stone has a different distribution of pores and it shines through the print. This is done to distinguish lithographs of dissimilar runs, because the porosity changes by changing the stone; but if we have the same run, the differences between each print are in the corrosion of the stone or in the various piles of color that is deposited on the paper when the print is made.

**Figure 3.**
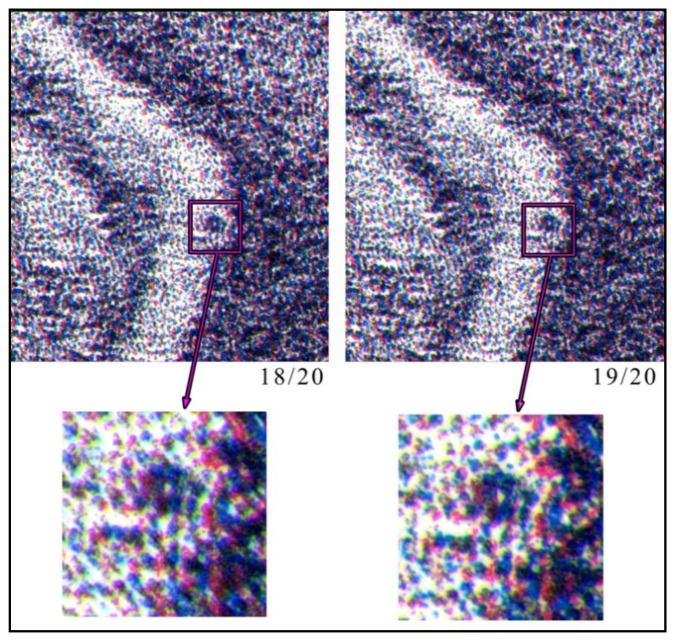
Particulars of two stone lithographs executed by the artist Giovanni Job (Dog 18/20 and Dog 19/20). One may see the slightly different distributions of colorful “stains”.

Also in this case there will be different distributions of colorful “stains”, similar to a speckle field. Figure 3 shows the same area of two different original copies of the same lithograph, the dog by Giovanni Job, which highlight how the two distributions are slightly different. This difference among similar copies is an important property, which allows using these distributions for uniquely identifying each artwork.

In addition to stone lithography there also exists the so called chalk-manner lithographs, produced using a wax crayon to draw an image onto a piece of limestone. The density of a line or shaded area directly corresponds to the amount of pressure applied while drawing; the slightly rough surface of the stone picks up more wax as pressure is increased. This ultimately produces an irregular stippled appearance in the final print. These features can be seen using relatively low magnification, such as the one obtained with a magnifier lens. In this paper we have considered only stone lithography, and used the stain distribution for our authentication procedure, having some stone lithography available for testing. In any case the procedure and the system, described in the following sections, is obviously applicable to any artwork made by different lithographical methods and to artworks in general, if a precise characterizing feature can be found (e.g., brush strokes).

Currently, the only method for certifying lithography authenticity is a Certificate of Authenticity and signature plus serial number directly impressed on the artwork itself (see Figure 2). The artist signs each impression as an approval if he considers it a good print. He signs his name in pencil along the lower right-hand corner of the paper. In addition, the author marks each lithograph with a number printed in the series and with the total number of prints in the edition.

It is important to notice that Certificate of Authenticity can be easily counterfeited, but also the serial number and signature, made by indelible pencil, can be perfectly reproduced using an autopen system [11,12].

By exploiting the potentiality offered by digital technologies, today new and more effective methods of authentication can be developed. In particular, a biometric-like approach can be used. Biometric identification relies on physical characteristics that are unique to each person to ascertain the identification of an individual. The most commonly known methods of biometric identification are fingerprints, DNA, iris scans, hand geometry, facial features, and voice. To translate this approach to artworks, it is necessary find unique, unrepeatable and unchangeable characteristics. In preceding works [13,14], this methodology has been named hylemetry.

### 2.2. Authenticity Certification Procedure

**Figure 4.**
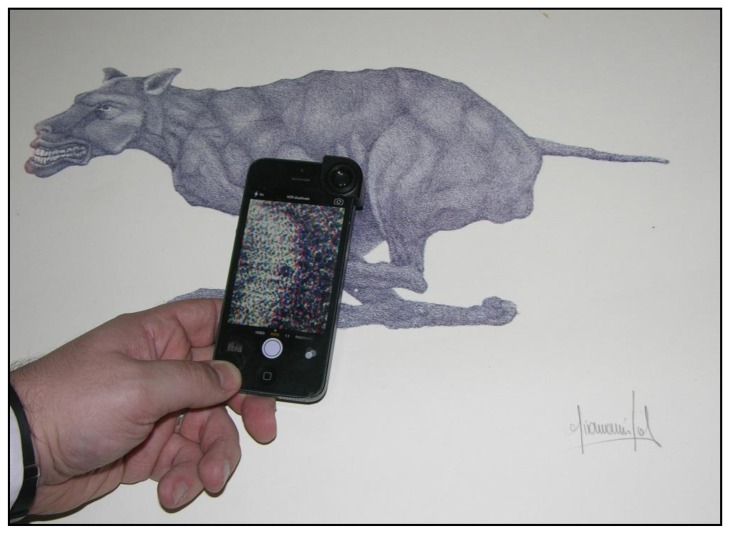
Example of smartphone used during acquisition.

To certify a lithograph's authenticity by means of hylemetric identification, it is necessary to acquire a unique, non-repeatable and immutable characteristic, as previously defined. In this paper, the non-repeatable and immutable characteristic is the colorful “stains”, acquired by means of a smartphone. Therefore, with the potentiality offered by modern smartphones referring to processor power and image elaboration, these can be easily transformed into excellent biometric (hylemetric) sensors [15]. The smartphone used in this paper is a common iPhone 5 equipped with an Olloclip^®^ 10× macro lens system. Figure 4 shows the smartphone system during the acquisition.

**Figure 5.**
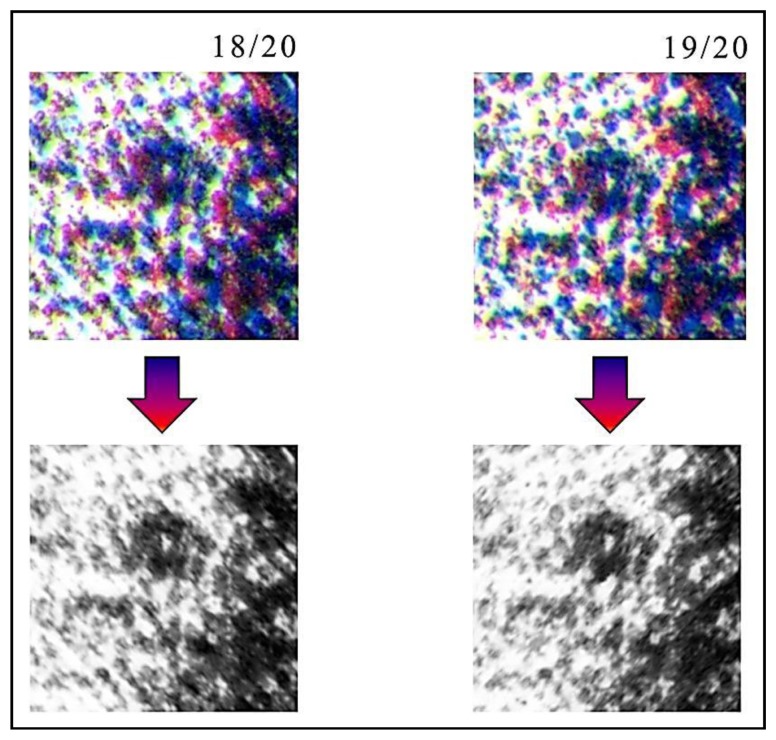
Hylemetric Hash Pattern of the Stone Lithography Dog 18/20 and Dog 19/20.

Subsequently, the colorful “stains”, acquired in RGB 24 bit format, are transformed to a uniform CIELAB color space [16]. After that, we use only the L channel, normalized with dynamic 0 to 1. In this way, we are sure that the obtained image is not affected by the environmental illumination. The obtained image has a typical speckle-like structure. This procedure is a one-way function, defined in the following as Hylemetric Hash Pattern (*HHP*). The Hylemetric Hash Patterns, extracted from the two Job's Dog Lithography, are shown in Figure 5.

Starting from the obtained *HHP*, the proposed authentication system wants to introduce a new digital certificate of authenticity, uniquely connected with a specific lithography using the *HHP* itself.

The author (or the certification authority) decides which part of the artwork has to be acquired. This is acquired at High Definition; in this way it is possible to extract the related *HHP*. The *HHP* is sent, with the artwork information and the author digital signature, to a centralized Artwork Digital Archive (ADA) server. The ADA software generates a unique artwork identification number and a dedicated Universal Resource Locator (URL), where the Digital Certificate is deployed. This process is similar to the digital object identifier (DOI) schema [17]. A DOI is a character string (a “digital identifier”) used for uniquely identifying an object such as an electronic document. Metadata about the object is stored in association with the DOI name and this metadata may include a location, such as a URL, where the object can be found.

In this case, the ADA sends back to the author (or to the certification authority) the artwork URL and the author can put it on the lithography itself (for example on its back) by means of a 2D barcode. For this last operation is important using a bonding based on an inert compound that does not degrade the artworks itself.

**Figure 6.**
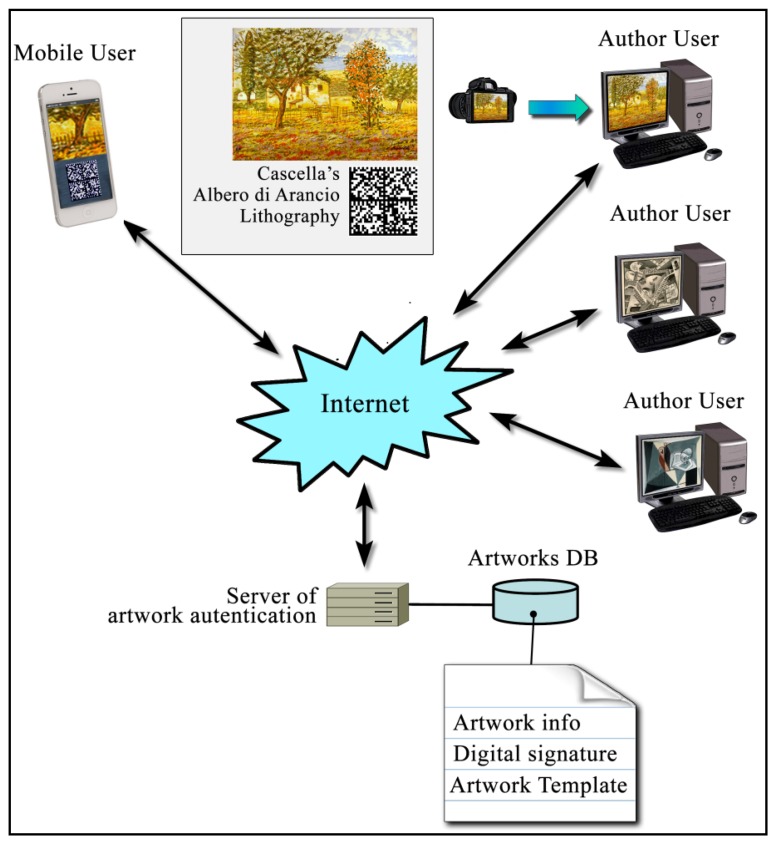
Schema showing the lithography authentication step.

In the URL, memorized in the barcode, will be possible insert all the lithography and author information, both technical and biographical, with the template (*i.e.*, the HHP and the information on the acquisition area), necessary to online authenticity verification. During this online verification, the smartphone, using a dedicated app (the common term for a smartphone application), acquires the barcode, decodes the content inside, goes to the indicated URL and retrieves the low definition image of the acquisition area, with the template. At this point, using the same app, the verifier acquires an area as similar as possible to the one reported in the low definition image, using the camera sensor available on the smartphone, and calculates the related *HHP*. At this point the smartphone app will be able to compare the two templates (the one retrieved by the ADA URL and the locally calculated one) and report the authenticity result. The schematic procedure is shown in Figure 6.

In order to extract a *HHP* good enough to compare with that retrieved using the ADA URL, it is necessary to correct any possible distortion and acquisition error before correlate the two images for verifying the lithograph authenticity.

Without a geometrical correction, it could be possible that the verifier may obtain a false negative result (*i.e.*, false lithography result in case of original one tested). To mitigate this situation, an Image Registration procedure it is necessary [18–23]. In our application, smartphone cameras have to capture images of flat objects (small area of lithographs). If we process only the central area of the images, neglecting the borders, where is always possible having distortions due to the camera itself, an automatic image registration can be implemented by means of Log-Polar—Transformation, Fourier—Transformation and Phase Correlation (*i.e.*, Fourier-Mellin Transform) [24,25].

### 2.3. Authenticity Verification App

To avoid copy attack, duplication, replacement of the template file (*i.e., HHP*), the use of a digital signature is necessary [26,27]. A digital signature guarantees that a document (in this paper the template constructed from the colorful “stains”) is original (*i.e.*, constructed by the artwork author or by the certifier company) and links the identity of the underwriter with the file and provides an official stamp (unalterable otherwise the digital signature verification fails) which legally determines the author of the document. These characteristics can be efficiently exploited to combat counterfeiting.

The template (*HHP*) is digitally signed by an asymmetric key algorithm, an encrypting “two keys system”, which exploits devices able to producing two different, but linked keys, one private (internal to the device and irretrievable) and the other public.

With the digital signature, we obtain *HHP_C_*; in this way, the data present in the certification media cannot be used for copy attack. Obviously, for verifying the lithograph originality, it is necessary, using the associated public key *k_pub_*, to decrypt the encoded information. The whole verification procedure can be implemented in an opportune application (app) that exploits the elaboration potentiality of smartphones. The smartphone app reads the 2D barcode, and extracts the ADA URL address. From the remote authentication archive, the smartphone retrieves the digital certificate of authenticity composed by the encrypted template *HHP_C_*, the public key *k_pub_* and a low definition image, indicating the area to be scanned. By means of public key *k_pub_*, we obtain *HHP_D_* from *HHP_C_*.

Subsequently, using the smartphone camera, an area as similar as possible to the one indicated in the digital certificate it is acquired, obtaining *I_S_*. The app is able now to calculate the related *HHP_S_*. To avoid effects due to geometric distortions, introduced during the acquisition step, an image registration procedure it is necessary on *HHP_S_*. Using *HHP_D_* and a Fourier-Mellin Transform on *HHP_S_* we obtain *HHP_R_* (acquired template with geometrical correction).

The Fourier- Miller Transformation automatically solves rotations, translations and scales, which are the most common errors introduced during the acquisition phases. We have used this kind of registration because our subjects are flat objects and, for avoiding any other image distortion (e.g., barrel), we have taken only the central part of the image itself (*i.e.*, 1,200 × 1,200 pixels, starting from an acquired image of 3,264 × 2,448 pixels). Obviously in case of 3D objects to be authenticated, a more sophisticated Image Registration will be necessary, to still allow an automatic procedure.

**Figure 7.**
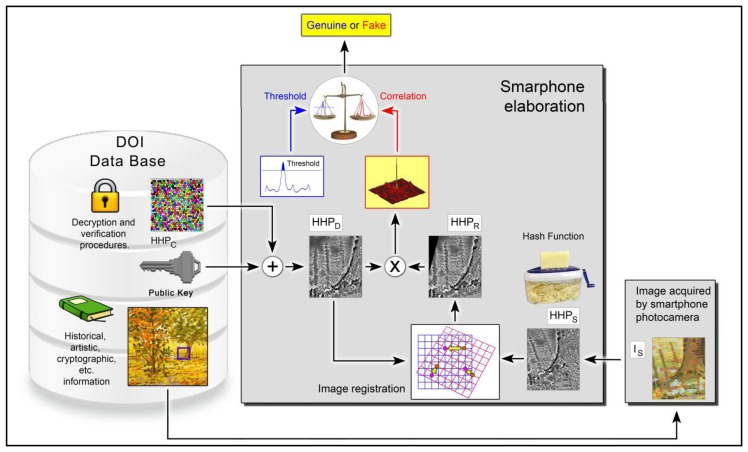
Complete schema showing the verification procedure app.

As the final step, *HHP_R_* is compared with *HHP_D_* to decide if the lithograph is original or not, respect to the data inserted in the ADA archive. Figure 7 shows the previously described process.

### 2.4. Verification Step

As previously stated, after having aligned the two templates, it is possible to verify if these are extracted from the same lithograph. In fact, the image transformation, made by means of Image Registration, only evaluates geometrical differences between the two *HHP* images. If we have two different lithographs having the same subject, the two images are at first sight similar, and after correcting for geometrical distortions, are pretty much the same image. The presence of randomly distributed hylemetric characteristics, such as the color pattern previously described, allows one to determine if the two images are extracted from the same lithograph or not. In fact, this random pattern is very different among similar artworks (see Figure 5) and a correlation approach applied to the corrected images can easily discriminate among similar lithographs.

It is important to underline that for verifying the similarity between the two images only geometrical position and shapes of color points are used (template is the L channel in the CIELAB color space).

Due to possible residual geometrical distortion after applying Image Registrations and the unavoidable presence of noise (*i.e.*, electronic noise, presence of dust on camera lens, resampling, numerical approximations), in this paper we propose a verification approach based on phase correlation method, similar to the one used in speckle field measurement [28]. The phase correlation between the decrypted template (*HHP_D_*) and the one obtained after geometrical correction (*HHP_R_*) allows to determine a correlation peak, translated from the center of two quantities equal to horizontal and vertical linear variances between the two images. The phase correlation surface is defined as [29,30]:

(1) $$C_{\alpha }=F^{-1}\left[\frac{F^{*}({\text{HHP}}_{D})F({\text{HHP}}_{R})}{{\left|F^{*}({\text{HHP}}_{D})F({\text{HHP}}_{R})\right|}^{\alpha }}\right].$$

In Equation (1) *F* and *F*^−1^ are forward and backward Fourier transform operators, respectively, and * represents the complex conjugate. Equation (1) is efficiently calculated using a Fast Fourier Algorithm. The coefficient *α* controls the correlation peak width. Optimum values range from *α* = 0 for image characterized by high spatial frequency content and high noise level, to *α* = 0 for low noise image with less fine structure. As proposed in literature [31,32], we have always used *α* = 0.5 values.

The phase correlation method provides a distinct sharp peak, whereas the classical cross correlation yields several broad peaks and a main one. A second important property is due to whitening of the signals by normalization, which makes the phase correlation notably robust to those types of noise that are correlated to the image function, e.g., uniform variations of illumination, offsets in average intensity.

Due to the impossibility that the template *HHP_R_* could be exactly the same referred with *HHP_D_*, we have defined a statistical verification threshold, *T*_α_ that has to be verified against the peak of the phase correlation surface *C*_α_(*peak*) so that:

(2) $$\{\begin{array}{cc} C_{\alpha }(\text{peak})<T_{\alpha } & \text{false lithography}\\ C_{\alpha }(\text{peak})\geq T_{\alpha } & \text{genuine lithography} \end{array}$$

The selection of the appropriate threshold is based on the minimization of False Acceptance Ratio, such as the percentage of a false lithograph recognized as true, respect the total amount of verification tests (it has to be noted that the introduction of geometrical correction has highly reduced False Rejection Ratio, due to genuine lithography no longer being recognized as counterfeited).

### 2.5. Performance of the Image Acquisition Media

The smartphone used in verification phase has to have proper characteristics to assure both barcode and HD image acquisition and their elaboration. Currently there exist a lot of apps dedicated to barcode acquisition and decoding and any smartphone is able to access the web to retrieve related information from an URL. On the contrary is not so easy find smartphones able to acquire, with an adequate resolution, the artwork texture. iPhone 5 or higher smartphones , as well as other high-level smartphones (e.g., Samsung S IV) can be adapted with external lens sets, to increase the internal camera performance. In this way, the smartphone camera can be transformed in an instrument able to capturing the details necessary for creating *HHP_S_*.

**Figure 8.**
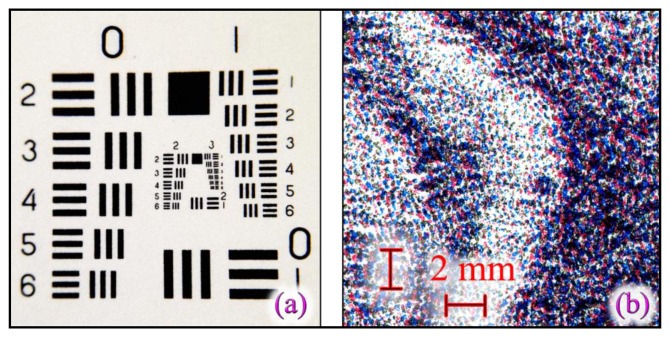
(**a**) SAF 1951 resolution target acquired with an iPhone 5 and an Olloclip^®^ macro 10× lens mounted on it. (**b**) Particular of Giovanni Job's Dog lithograph acquired under the same conditions.

In our experiments, we have used an Apple^©^ iPhone 5 equipped with an Olloclip^®^ macro 10× lens, able to acquire an area of about 15 × 15 mm with a resolution better than 14 lines per mm (∼350 dpi). Figure 8a shows the USAF 1951 resolution target acquired by the iPhone 5 with Olloclip^®^ macro 10× lens used. Figure 8b shows a lithograph particular, captured under the same conditions. From these figures is easy to understand that the smartphone used has the ability to acquire the necessary textures used in the non-cloneable *HHP* creation. In fact, the typical lithography structure has color stains with dimensions more than 0.1 mm, which allows defining a necessary acquisition resolution better than 250 dpi.

## 3. Safeart System

**Figure 9.**
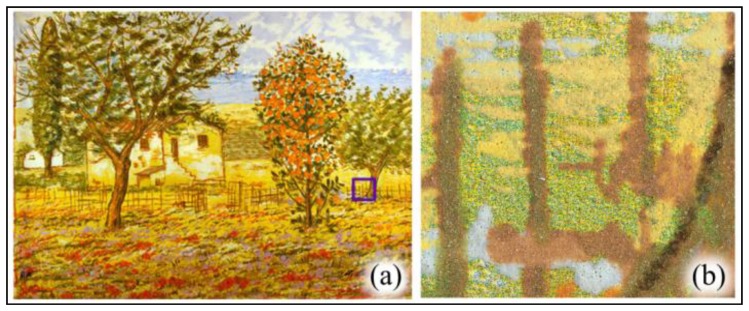
Cascella's Albero di Arancio lithograph. (**a**) Lithograph with the zone used for certification highlighted. (**b**) Portion of the lithography acquired by a smartphone (*I_S_*).

In this section some experimental results are reported. Figure 9 shows Cascella's lithograph Albero di Arancio. In our experiments, we used this particular lithography because it was the subject of forensic analyses in which we were involved as investigators. Figure 9a shows the lithograph with the zone used for certification highlighted. In Figure 9b the authentication zone acquired by means of the smartphone (*I_S_*) is shown.

**Figure 10.**
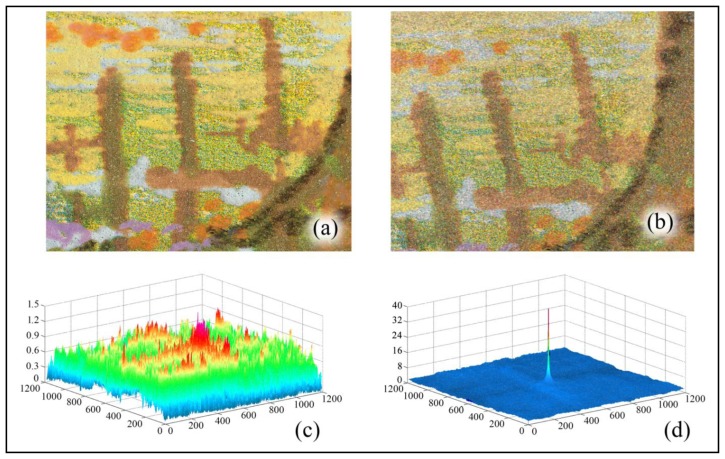
Example of verification result. (**a**) Certificate Image; (**b**) Roto-traslated acquired image with Gaussian Noise added; (**c**) Correlation result without Image Registration; (**d**) Correlation result after Image Registration.

It is easy to understand that the manual acquisition of the image can lead to different scales, rotations and translations with respect to the image used in the certification media. Figure 10 shows the image used in the certification media *vs*. a typical acquired image.

To verify the robustness of the proposed system, we have digitally applied Salt and Pepper noise to the acquired image, which degrades the resulting *C_α_*(*peak*) values; in any case, the resulting value is still over the threshold *T_α_*. In Figure 10c,d are also drawn *C_α_* with and without geometrical transformation, to highlight how significantly it changes in the two cases. Table 1 reports the phase correlation peak value *C_α_*(*peak*) for the different added noise and the related necessary parameters. There are also reported in the different statistical threshold values for a set of possible thresholds.

It could be noted that the used threshold vary in any experiment. This is due to the fact that we have used an adaptive statistical threshold, based on statistical characteristics of the correlation function. The threshold *T_α_* used in this article is:

(3) $$T_{\alpha }=3\cdot {\bar{m}}_{C}$$

where *m̄_C_* is the mean value of *C_α_*. It has to be noted that it is possible to define alternative statistical thresholds. We have also used in our experiments different thresholds, with similar results, such as three times the mean value plus once standard deviation, three times the standard deviation plus once mean value and so on. Table 1 also shows results for these different thresholds. The choice of the threshold in Equation (3) is due to the very low variance of it values among different noises. For avoiding misunderstanding, the variance and mean value described in relation to Gaussian Noise are the noise statistical values, not directly related to the mean value and standard deviation present in the heading row, which are, as previously described, statistical values extracted from the correlation function.

**Figure 11.**
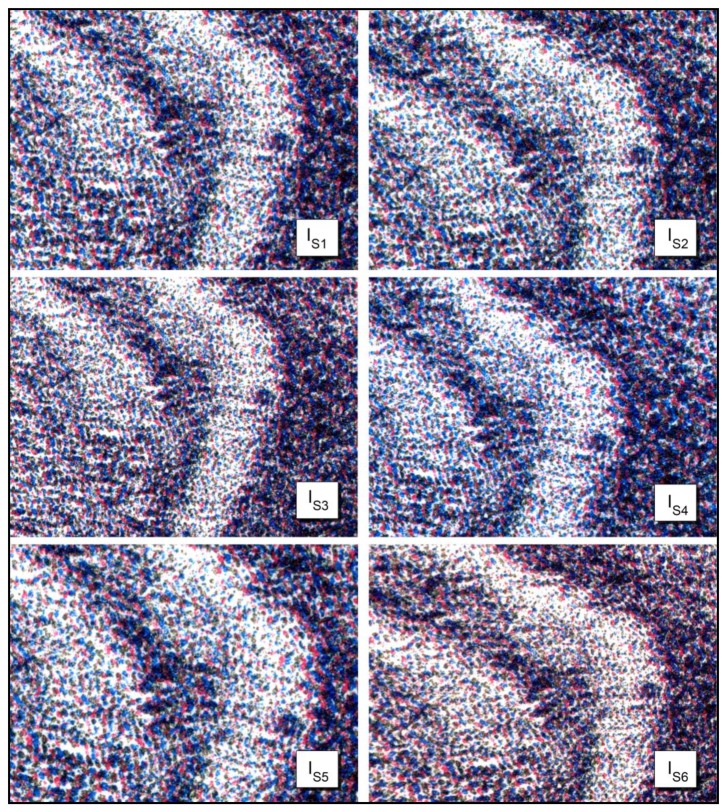
Job's lithograph captured under different illumination conditions (particular of Dog 18/20).

Figure 11 shows six different image acquisitions obtained from the same Job lithography Dog 18/20. These acquisitions are carried under six different illuminations. As reported in Table 2, the use of the L channel in the CIELAB color space, allows the correlation coefficient to be independent from the acquisition environment.

**Figure 12.**
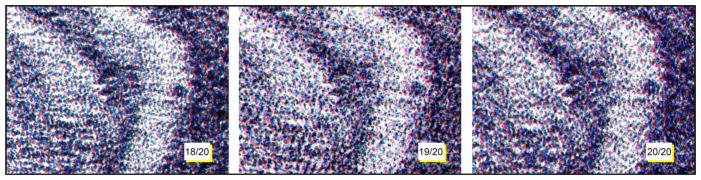
Lithograph particular captured from three different copies of the same lithography.

In Figure 12, three different lithographs of the same series (Giovanni Job's Dog 18/20, 19/20 and 20/20), are shown. Applying the system to them, it is demonstrated that they are different, even if the reported particulars could seem identical. This difference is due to the technique used to create a stone lithograph: to obtain color lithography, three subsequent impressions are made, using three different stones. It is impossible to place the three stones in exactly the same position; therefore, each copy is slightly different from the others. Then, using as hylemetric characteristic the positions of the colorful stains, the system is able to identify them as different copies. In this case, we obtained low *C_α_*(*peak*) values (compared with the ones obtained in case of using the same lithography): *C_α_*(*peak*) = 2.01 in case of lithography 18/20 compared with 19/20; *C_α_*(*peak*) = 2.76 in case of lithography 18/20 compared with 20/20; and *C_α_*(*peak*) = 2.46 in case of lithography 19/20 compared with 20/20. In these tests, according with Equation (3) we have a verification threshold equal to 3.55.

This reported case can be associated with an illegal copy of the original artwork, verified using a copy of the original Digital Certificate. The private key has allowed to extract *HHP_D_*, but the correlation with the calculated *HHP_S_* is lower that the threshold, also reported in the digital certificate. A simple connection to the ADA URL, retrieved from the 2D barcode, has allowed us to certify the non-originality of the artwork or, at least the absence of evidence of originality (*i.e.*, an own Digital CoA).

## 4. Conclusions

Art authentication is an increasingly fraught field, with artist-specific foundations, collectors and experts tangled over who has the final word over attribution. In this paper, we have developed a simple, robust, efficient and low-cost system for lithography authentication. We have used known methodologies, normally applied in other technical fields, such as image registration in medicine, surface phase correlation in mechanical engineering, and authentication in biometry. In any case, their use for the authentication of artworks is innovative.

In parallel with the acquisition and verification procedure, we have also proposed the use of a remote Database (Artwork Digital Archive), similar to the DOI one. For extensive application, the Artwork Digital Archive would have to be managed by a third part authority, similar to the “Database of Stolen Artworks”, managed by Italian Carabinieri division for the Protection of Cultural Heritage, or “National Stolen Art File (NSAF)”, managed by the U.S. F.B.I.

We have tested our system using a simple iPhone 5. Obviously, any other high-end device can be used (e.g., Samsung Galaxy S family, Nokia Lumia family). Our system works well on stone lithographs. Future work will be carried out to verify our method, even on fine art digital copies (e.g., in offset lithography) [33].

The authentication procedure uses a biometric-like (hylemetric) approach. This methodology can be easily applied, also, for other inert matter, such as pharmaceutical packaging, identification documents and so on [34,35].

We propose an image processing procedure that is easily implemented as a smartphone app, but using high performance tablets, more sophisticated authentication procedures can be achieved. In particular, Image Registration can be used [36], able to work with distorted images, and/or multiple templates [37], similar to what is done in medical diagnostics to significantly reduce false positive cases.

## Figures and Tables

**Table 1.** Correlation values for different added noise, with related noise parameters. It has to be noted that statistical thresholds are constructed using *correlation function mean value* and standard deviation and are not directly connected to any noise function mean values and variances.

**Noise Type**	**Noise Parameters**	3*m̄_C_* + *σ_C_*	3*m̄_C_*	3*σ_C_* + *m̄_C_*	$3\sqrt{\sigma_{C}}$	$3\sqrt{\sigma_{C}+{\bar{m}}_{C}}$	*C_α_*(*peak*)
Gaussian	Mean Value equal to 0.10	4.09	3.07	4.09	3.03	4.29	31.04
	Variance equal to 0.10						
Gaussian	Mean Value equal to 0.10	3.99	3.03	3.88	2.93	4.20	28.81
	Variance equal 0.15						
Gaussian	Mean Value equal to 0.15	4.10	3.09	4.04	3.00	4.28	30.47
	Variance equal to 0.10						
Gaussian	Mean Value equal to 0.15	4.00	3.07	3.84	2.90	4.20	28.38
	Variance equal 0.15						
Salt & Pepper	Distribution equal 0.1	4.15	3.01	4.42	3.20	3.39	34.81
Salt & Pepper	Distribution equal 0.2	4.01	2.96	4.11	3.06	4.28	31.60
Salt & Pepper	Distribution equal 0.3	3.86	2.92	3.80	2.91	4.15	28.30
Poisson	No parameter to be defined	4.21	3.04	4.52	3.24	4.53	35.90
Speckle	Distribution equal to 1.0	3.53	2.79	3.15	2.58	3.87	22.29
Speckle	Distribution equal to 1.5	3.42	2.75	2.92	2.45	3.78	20.07
Speckle	Distribution equal to 2.0	3.34	2.73	2.73	2.34	3.70	18.42

**Table 2.** Correlation values *C_α_*(*peak*) for the images shown in Figure 11.

	***I***_***S***1_	***I***_***S***2_	***I***_***S***3_	***I***_***S***4_	***I***_***S***5_	***I***_***S***6_
*I*_***S***1_	**42.00**	33.07	34.09	33.03	34.29	32.59
*I*_***S***2_	33.99	**42.00**	33.88	32.93	34.20	33.91
*I*_***S***3_	34.10	33.09	**42.00**	33.00	34.28	33.20
*I*_***S***4_	34.00	33.07	33.84	**42.00**	34.20	34.93
*I*_***S***5_	34.15	33.01	34.42	33.20	**42.00**	32.85
*I*_***S***6_	34.01	32.96	34.11	33.06	34.28	**42.00**
